# Impact of Bottle Type and Gelatin-Based Film Strips with Ethyl Sinapate on Oxidative Status and Antioxidant Properties of Cold-Pressed Rapeseed Oil

**DOI:** 10.3390/foods15010046

**Published:** 2025-12-23

**Authors:** Dobrochna Rabiej-Kozioł, Alicja Tymczewska, Aleksandra Szydłowska-Czerniak

**Affiliations:** Department of Analytical Chemistry and Applied Spectroscopy, Faculty of Chemistry, Nicolaus Copernicus University in Toruń, Gagarina 7, 87-100 Toruń, Polandolasz@umk.pl (A.S.-C.)

**Keywords:** antioxidant-active films, phenolic acid ester, oxidative stability, vegetable oil

## Abstract

Cold-pressed rapeseed oil aligns well with the trend of growing demand for minimally processed, health-promoting food products. It is essential to identify suitable storage conditions that protect cold-pressed rapeseed oil from oxidation, thereby extending its shelf life. In this study, the effect of gelatin/polyvinyl alcohol film strips enriched with ethyl sinapate (GPE) and immersed in cold-pressed rapeseed oil samples was evaluated during an accelerated storage test (14 days at 40 ± 1 °C under light (power of luminous flux = 385 lm). The influence of bottle type differing in shape (Marasca and Dorica) and glass colour (amber and clear) was also assessed. The incorporation of GPE into the stored oils enhanced their antioxidant activity (AA) determined by 2,2′-azino-bis(3-ethylbenzothiazoline-6-sulfonic acid (ABTS = 1956.78–2334.10 µmol Trolox (TE)/100 g), 2,2-diphenyl-1-picrylhydrazyl (DPPH = 528.29–691.19 µmol TE/100 g), ferric reducing antioxidant power methods (FRAP = 454.14–511.61 µmol TE/100 g) and total phenolic content (TPC = 41.62–47.25 mg sinapic acid (SA)/100 g) compared to oils without film strips (ABTS = 1217.89 –1422.80 µmol TE/100 g, DPPH = 376.85–464.13 µmol TE/100 g, FRAP = 98.28–126.40 µmol TE/100 g and TPC = 6.38–8.02 mg SA/100 g) after first week of storage and confirmed the effective gradual release of ethyl sinapate from films to oils during two weeks of accelerated storage (ABTS = 2064.80–3086.47 µmol TE/100 g, DPPH = 597.11–854.37 µmol TE/100 g, FRAP =428.00–599.76 µmol TE/100 g, and TPC = 35.02–57.19 mg SA/100 g). Moreover, the GPE inhibited oil deterioration by reducing both primary (peroxide value (PV) = 3.75–5.11 meq O_2_/kg and 3.64–4.89 meq O_2_/kg, K_232_ = 1.236–1.494 and 1.551–1.675 after the first and second week of storage, respectively) and secondary oxidation products (anisidine value (pAnV) = 1.03–1.16 and 1.08–1.61; K_268_ = 0.102–0.170 and 0.185–0.237 after the first and second week of storage, respectively) compared to oxidative status of oils without film strips (PV = 3.76–5.59 meq O_2_/kg, K_232_ = 1.452–1.828, pAnV = 0.85–2.27, K_268_ = 0.154–0.263). In addition, synchronous fluorescence spectroscopy was applied to monitor changes in the main fluorescent components of the studied oils. Overall, the use of a dark glass bottle combined with antioxidant film strips proved to be an effective strategy for prolonging the shelf life of cold-pressed rapeseed oil.

## 1. Introduction

Vegetable oils, including rapeseed oil, are key components of a healthy diet. Rapeseed oil is the third most commonly consumed edible oil worldwide, with domestic consumption estimated at 33.96 million metric tons for the 2024/25 season [1]. Notably, cold-pressed rapeseed oil contains higher concentrations of bioactive compounds beneficial to human health, such as vitamins A and E, phytosterols, carotenoids, and polyphenols, compared with refined oils. Therefore, from a nutritional point of view, the consumption of cold-pressed oils is preferred [2]. The European cold-pressed oils market generated revenues of USD 4848.8 million in 2024 and is projected to reach USD 6590.2 million by 2030 [3].

Cold-pressed rapeseed oil is characterized by a high content of unsaturated fatty acids, mainly oleic acid (58–62%) and polyunsaturated fatty acids (PUFAs) (28.1 g/100 g), including α-linolenic acid (9.1 g/100 g), an omega-3 fatty acid. It is also rich in vitamins (A, D, E, K), sterols, polyphenols, tocopherols, and β-carotene. Since Western diets often lack sufficient omega-3 from fish, cold-pressed rapeseed oil serves as a valuable alternative source [2,3,4,5]. However, the high PUFA amounts make it prone to lipid oxidation, which can deteriorate sensory quality and potentially have a negative impact on health [4].

To improve oil quality and oxidative stability, several approaches, such as adding antioxidants or optimizing storage conditions, can be employed. Synthetic antioxidants (e.g., butylated hydroxyanisole-BHA, butylated hydroxytoluene-BHT) effectively inhibit lipid oxidation but their use is restricted due to potential toxic and carcinogenic effects [6,7,8]. Consequently, growing consumer interest in natural, minimally processed products has led to a shift in research toward natural antioxidants.

Unfortunately, only a small fraction of natural antioxidant compounds is transferred from rapeseed into the cold-pressed oil during mechanical pressing. Moreover, the low solubility of hydrophilic polyphenols in lipophilic media limits their direct use in oils. To overcome this limitation, numerous studies have demonstrated that lipophilization of phenolic acids, through esterification with alkyl chains, yields amphiphilic molecules with improved antioxidant efficiency in oils [6,8]. Naturally occurring short-chain esters of hydroxycinnamic acids have high antioxidant activity and low toxicity in vitro, supporting their potential use as food additives [9,10,11]. Previous studies have shown that short-chain alkyl esters of hydroxycinnamic acids (e.g., methyl and ethyl derivatives of caffeic, ferulic, and sinapic acids) exhibit protective effects on vegetable oils.

More recent findings indicate that active packaging materials incorporating antioxidants are even more effective than direct supplementation. This improvement is attributed to the controlled release of active compounds, lower quantities required, and direct surface contact with the food (in the case of products with a solid consistency), as well as simplifying the production process by eliminating the step of adding an antioxidant. It is essential to note that the controlled release of antioxidants, as opposed to their direct and immediate incorporation into the product, enables the maintenance of a constant antioxidant concentration in the food, allowing for continuous compensation of antioxidant depletion during storage. Furthermore, some antioxidants may exhibit pro-oxidant activity when their concentration exceeds a critical threshold. Therefore, controlled delivery is crucial to ensure effective inhibition of oxidation [12]. Active packaging is regulated under EU legislation (EC No. 1935/2004 [13] and EC No. 450/2009 [14]) and is defined as packaging that “incorporates components that release or absorb substances into or from the packaged food or the surrounding environment,” with the goal of extending shelf life and maintaining product quality [15]. Recently, natural antioxidants such as extracts from green tea, rosemary and oregano, tocopherol, and carvacrol, as well as phenolic acids (e.g., ferulic, gallic, and cinnamic acids), have been extensively studied as packaging additives [12,16]. Their incorporation not only delayed oxidation but also modified the physicochemical properties of the packaging material. Meanwhile, caffeic acid added to chitosan–fish gelatin films enhanced mechanical strength through cross-linking, while ferulic acid improved thermal stability via hydrogen bonding with chitosan. It is noteworthy that the active package could be used in two ways: as an independent packaging material by incorporating active compounds in the wall of the package and compounds absorbing undesirable compounds from food and headspace, or releasing desirable compounds from the wall, or by addition to a conventional “passive” package sachet, pad or label with active compounds [17]. Despite the growing interest in active packaging, there remains a need for innovative solutions tailored to specific products and their antioxidant protection requirements. In this context, the present study introduces a novel concept by designing an amphiphilic antioxidant-loaded gelatin/polyvinyl alcohol film to inhibit oxidation in vegetable oil. The proposed film is edible and safe because it is made from materials registered as food additives, including polyvinyl alcohol (E1203) and gelatin (E428). Although ethyl sinapate is not on the EU list of authorized food additives, our previous study confirmed its safety [18]. Additionally, other compounds belonging to phenolic acid esters are included in the list E310 (propyl gallate).

Nevertheless, environmental considerations are important in the development of new packaging. Consequently, the selection of environmentally friendly materials becomes a key priority. Therefore, the chosen material should be biodegradable, inexpensive, and have minimal or no release of pollutants during the production process. Considering both environmental and economic factors, the gelatin/polyvinyl alcohol composition produced by the solvent casting method has been widely used as a matrix for incorporating various bioactive additives [19]. However, scaling up to industrial production of active films requires careful consideration of process costs and efficiency due to the price of the active antioxidant components added to polymer matrices [20]. Additionally, introducing the immersion film to standard packaging does not change the functional properties of the well-known and already approved consumer package.

Finally, when designing packaging, attention should also be given to its appearance and materials, as these factors influence consumer perception and expectations of product quality by eliciting emotional responses [21]. For this reason, the direct use of biodegradable or edible films may not fully meet the expectations of premium oil consumers.

On the other hand, appropriate storage conditions, including packaging, are crucial for maintaining the quality of oils (in terms of colour, aroma, taste, and nutritional value) from production to consumption. Glass, regardless of its colour (clear, dark, or opaque), is one of the most widely used packaging materials for olive and cold-pressed oils. The ability to produce bottles of various shapes, colours, and decorative finishes enhances consumer perception of oils sold in glass as premium products. Moreover, glass packaging offers several advantages, including environmental benefits due to its reusability and excellent protection against oxidation, owing to its impermeability to gases, in contrast to undesirable compounds from food and headspace or releasing desirable compounds from wall, or by addition to conventional “passive” package sachet, pad or label with active compounds polyethylene terephthalate (PET) container, which only slow down O_2_ exchange [22,23,24]. However, limiting light transmission through oil bottles—which induces photo-oxidation—remains a challenge for both glass and PET containers. The protective effect of a bottle against light depends primarily on wall thickness and colour.

Each packaging material has advantages and limitations; thus, it must be carefully selected and tested for a given product. Consequently, the present study aimed to investigate the influence of active gelatin/polyvinyl alcohol film strips loaded with ethyl sinapate (GPE) applied to different glass bottle types (varied in shape and colour) on the antioxidant activity (AA), total phenolic content (TPC), and oxidative stability of cold-pressed rapeseed oil during accelerated storage simulating actual storage environments in retail places or households. Quality changes in the stored oils under accelerated conditions were also evaluated by fluorescence spectroscopy.

## 2. Materials and Methods

### 2.1. Chemicals and Oil Samples

#### 2.1.1. Chemicals

All chemicals used in this study were of analytical or HPLC grade and purchased from Alchem (Toruń, Poland). Redistilled water was used for the preparation of all solutions.

#### 2.1.2. Cold-Pressed Rapeseed Oil

Rapeseed (*Brassica napus* L.) was kindly supplied by a local vegetable oil manufacturer. The oil was mechanically extracted using a Komet Screw Oil Expeller CA 168 59 G—CA 59 G3 (Economic Society “MEGART” Sp. z o.o., Katowice, Poland) powered by a 1.1 kW three-phase electric motor. Oil pressing was performed using a screw shaft with a 6 mm nozzle diameter. The temperature of the expressed oil was monitored using a non-contact portable infrared thermographic camera (Flir C5, Teledyne FLIR Fire & Rescue, Warszawa, Poland).

### 2.2. Sample Preparation

#### 2.2.1. Preparation of Active Film

The synthesis of ethyl sinapate by a modified Fischer esterification method was described in our previous work [7].

The active film was also prepared according to the methodology reported previously, with slight modifications. Briefly, 50 mL of 5% (*w*/*v*) gelatin solution, 30 mL of 5% (*w*/*v*) polyvinyl alcohol solution, and 3 mL of glycerol were mixed with 10 mL of ethyl sinapate (previously dissolved in ethanol) and 7 mL of redistilled water under magnetic stirring at 60 °C for 20 min. The resulting mixture was then sonicated for 3 min to remove air bubbles. Subsequently, the solution of gelatin/polyvinyl alcohol with ethyl sinapate was poured into Petri dishes and left to dry at room temperature. The obtained film was cut into strips measuring 70 mm × 10 mm. The obtained foil was described in detail and analyzed in our previous work [7].

#### 2.2.2. Accelerated Storage Test of Cold-Pressed Rapeseed Oil

The freshly cold-pressed rapeseed oil was poured into 250 mL Marasca and Dorica bottles made of clear and dark glass (four bottles of each type). Four strips of the prepared active film were immersed in half of the bottles.

**Figure 1:**
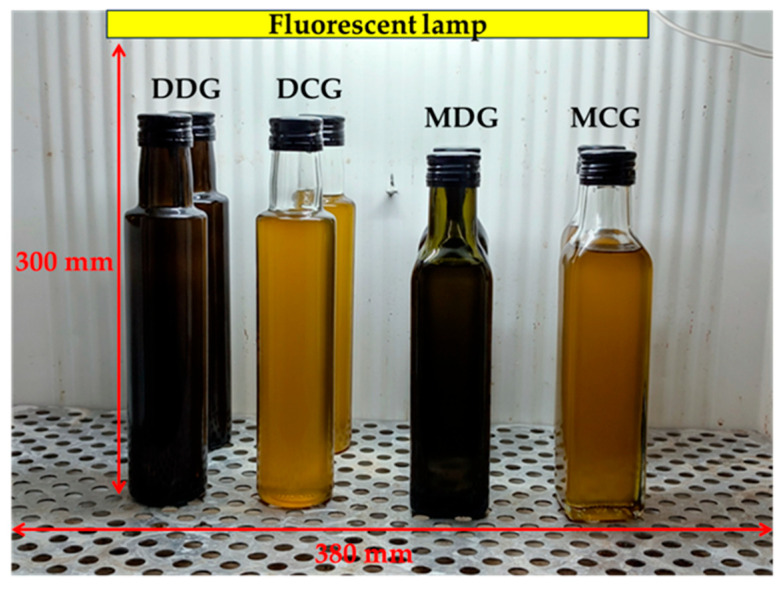
Position of bottles containing cold-pressed rapeseed oil without and with gelatin/polyvinyl alcohol film strips incorporating ethyl sinapate in an incubator during shelf-life test.

The accelerated storage test was performed in accordance with the protocol detailed in our previous work [7]. Briefly, the storage test was divided into two parts due to the incubator capacity (eight bottles). Eight bottles, with and without the active film strips, were placed in an incubator (Elkon CWE-2a, Łódź, Poland) at 40 °C under fluorescent light (T5 8 W F8W/33 GE, luminous flux = 385 lm, wavelength = 380 nm). The lamp was positioned 300 mm above the incubator shelf. The arrangement of bottles in the incubator is presented in Figure 1.

The experiment was conducted over a period of two weeks, during which one week of storage under these conditions was considered equivalent to approximately three months of storage under standard conditions. These described accelerated conditions mimic the autoxidation process of cold-pressed rapeseed oil under real storage conditions, defining its 6-month shelf life period.

Therefore, this experiment provided 17 cold-pressed rapeseed oil samples, including fresh cold-pressed rapeseed oil (O) and oil samples stored in: clear Dorica glass bottles without (O (DCG)) and with active GPE film strips (O+GPE (DCG)), dark Dorica glass bottles without (O (DDG)) and with active GPE film strips (O+GPE (DDG)), clear Marasca glass bottles without (O (MCG)) and with active GPE film strips (O+GPE (MCG)), and dark Marasca glass bottles without (O (MDG)) and with active GPE film strips (O+GPE (MDG)) for 1 and 2 weeks, respectively.

### 2.3. Analysis of the Oxidation Status of Cold-Pressed Rapeseed Oils

The characteristic parameters of the oil corresponding to primary and secondary oxidation products were determined according to standard ISO methods. The peroxide value (PV), indicating the formation of primary oxidation products, was analyzed according to ISO 3960:2017 [25], while the p-anisidine value (pAnV), corresponding to secondary oxidation products, was determined according to ISO 6885:2016 [26].

The overall oxidation state of the oil (TOTOX value) was calculated using the equation: TOTOX = 2 × PV + pAnV

The acid value (AV), reflecting the degree of oil hydrolysis, was determined following the ISO 660:2020 method [27].

Additionally, changes in oil absorption in the ultraviolet (UV) region were monitored to assess the formation of conjugated dienes (K_232_) and conjugated trienes (K_268_). The absorbances of oil samples dissolved in n-hexane (1% solutions) were measured at 232 nm and 268 nm, respectively, using a 1 cm quartz cuvette and a UV–Vis spectrophotometer (U-2900, Hitachi High-Technologies Corporation, Tokyo, Japan), in accordance with ISO 3656:2011 [28].

### 2.4. Analysis of Antioxidant Properties of Cold-Pressed Rapeseed Oils

Methanolic extracts of oil samples were prepared to evaluate the antioxidant properties, following a previously described procedure [7]. Briefly, 2.00 g of each oil sample was extracted with 5 mL of methanol using a laboratory shaker (SHKA 2508-1CE, Labo Plus, Warszawa, Poland) for 30 min. The mixtures were subsequently stored in a freezer to allow phase separation. Each extraction was performed in triplicate, and the resulting extracts were combined and stored at 4 °C until analysis.

The AA and TPC were determined using modified spectrophotometric methods: 2,2′-azino-bis(3-ethylbenzothiazoline-6-sulfonic acid (ABTS), 2,2-diphenyl-1-picrylhydrazyl (DPPH), ferric reducing antioxidant power (FRAP), and Folin–Ciocalteu (F-C), as described in our previous work [29]. All absorbance measurements were performed using a UV–Vis spectrophotometer (U-2900, Hitachi, Tokyo, Japan) with a 1 cm quartz cell.

ABTS, DPPH, and FRAP results are presented as µmol Trolox equivalents per 100 g of oil, and TPC is given as mg sinapic acid (SA) equivalents per 100 g of oil.

#### 2.4.1. ABTS Assay

The ABTS^•+^ solution was prepared by mixing a 7 mmol/L ABTS solution with a 2.45 mmol/L potassium persulfate solution (2:1). The prepared mixture was kept in the dark for 16 h. Then, the ABTS^•+^ mixture was diluted with ethanol to achieve an absorbance of 0.700 ± 0.020 at 734 nm. To measure the ABTS radical scavenging activity of the oil sample, 0.1–0.2 mL of the oil extract was added to 2.4–2.3 mL of the ABTS^•+^ solution, and the mixture was incubated at 37 °C for 1 min. The absorbance was subsequently measured at 734 nm using a reagent blank consisting of 2.5 mL of the ABTS^•+^ solution. The calibration curves were generated using working solutions of Trolox (TE) in methanol at concentrations ranging from 0.03 to 0.15 µmol/mL. The resulting calibration equation was:

$$\%ABTS=\left(320.7\pm 11.0\right)xc_{TE}+(3.22\pm 0.96)\;(R^{2}=0.9919).$$

#### 2.4.2. DPPH Assay

Briefly, 0.10–0.25 mL of the oil extracts were mixed with 1.90–1.75 mL of methanol, respectively. Then, 0.50 mL of a DPPH methanolic solution (304.0 µmol/L). The mixtures were shaken thoroughly and kept in the dark for 15 min. Afterwards, the absorbance was measured at 517 nm using a reagent blank consisting of 2 mL of methanol and 0.5 mL of the DPPH solution. The calibration curves were generated using working solutions of Trolox in methanol at concentrations ranging from 0.002 to 0.010 µmol/mL. The resulting calibration equation was:

$$\%DPPH=\left(611.1\pm 14.3\right)xc_{TE}\;+(4.88\pm 0.93),\;(R^{2}=0.9962).$$

#### 2.4.3. FRAP Assay

A freshly prepared FRAP reagent (2.5 mL of 10 mM TPTZ in 40 mM HCl, 2.5 mL of 20 mM FeCl_3_, and 25 mL of 0.1 M acetate buffer (pH 3.6)) was incubated at 37 °C for 10 min. Then, 0.15–0.40 mL of methanolic extract of oil sample and 2 mL of the FRAP reagent were transferred into a 10 mL volumetric flask and diluted to volume with redistilled water. The resulting blue solutions were kept for 20 min, and then the absorbance was measured at 593 nm against reagent blank. The calibration curves were generated using working solutions of Trolox in methanol at concentrations ranging from 0.002 to 0.010 µmol/mL. The resulting calibration equation was:

$$A_{593}=\left(37.66\pm 1.18\right)xc_{TE}+(0.218\pm 0.022),\;(R^{2}=0.9949).$$

#### 2.4.4. Folin–Ciocalteu Assay

Briefly, 0.5–2.0 mL of oil extract and 0.5 mL of F-C reagent were transferred into 10 mL volumetric flask. The obtained mixture was mixed thoroughly and left for 3 min. Then, 1 mL of saturated sodium carbonate solution (22.0%) was added, and the volume was adjusted to the mark with redistilled water. After keeping the solution in a dark place for 1 h, the mixture was centrifuged for 15 min (MPW-150R centrifuge, MPW MED. INSTRUMENTS, Warszawa, Poland), and absorbance was measured at 765 nm against a reagent blank. The calibration curves were generated using working solutions of sinapic acid in methanol at concentrations ranging from 0.15 to 2.5 mg/mL. The resulting calibration equation was:

$$A_{765}=\left(63.94\pm 2.16\right)xc_{SA}+(0.053\pm 0.0018),\;(R^{2}=0.9952).$$

### 2.5. Fluorescence Study

Fluorescence analysis was conducted in the same manner as described in our previous work [7]. Synchronous fluorescence (SF) spectra of cold-pressed rapeseed oils diluted in n-hexane were recorded over the 200–800 nm range with a 2 nm resolution, and an offset (Δλ) of 10 nm between excitation and emission monochromators. All spectra were acquired using a fully computerized spectrofluorometer (fluoroSENS, Gilden Photonics Ltd., Glasgow, UK) equipped with a xenon lamp.

### 2.6. Statistical Analysis

All measurements were performed in triplicate, and results were expressed as mean ± standard deviation (SD). The principal component analysis (PCA) and hierarchical cluster analysis (HCA) were performed by the Statistica 8.0 software (StatSoft, Tulsa, OK, USA), whereas the PAST programme was applied to assess correlations among oxidation parameters and antioxidant properties of the studied oil samples. Differences between mean values were assessed using one-way ANOVA followed by Duncan post hoc test at a significance level of *p* < 0.05 (different letters indicate significant differences).

## 3. Results and Discussion

### 3.1. Changes in Oxidative Status of Cold-Pressed Rapeseed Oils During Accelerated Storage

**Figure 2:**
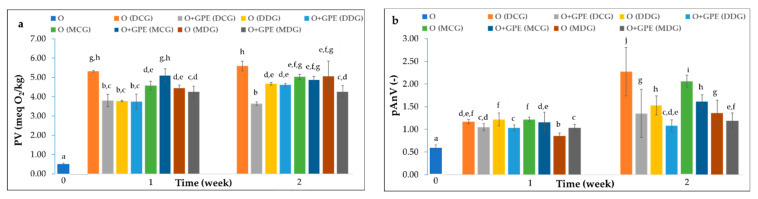

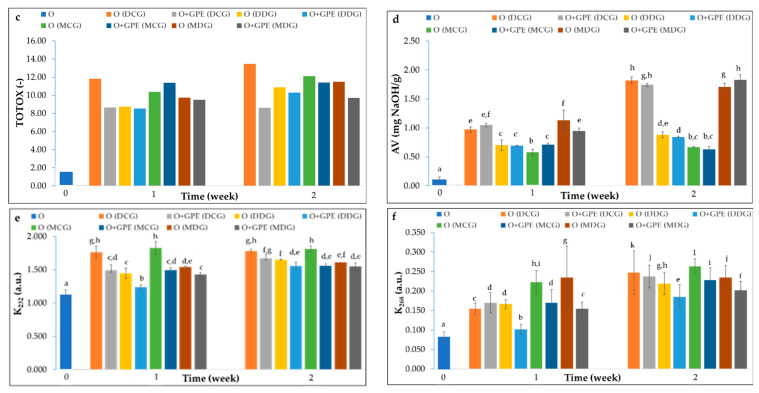
Changes in the oxidative status: PV (**a**), pAnV (**b**), TOTOX (**c**), AV (**d**), K_232_ (**e**), and K_268_ (**f**) results of cold-pressed rapeseed oils (O) without and with gelatin/polyvinyl alcohol film strips loaded with ethyl sinapate (GPE) stored for two weeks under accelerated conditions in Dorica clear glass bottles (DCG), Dorica dark glass bottles (DDG), Marasca clear glass bottles (MCG), and Marasca dark glass bottles (MDG). Bars with different letters indicate significant differences (one-way ANOVA and Duncan test, *p* < 0.05).

Figure 2 presents the degradation rates of the investigated oils, as assessed by the PV, pAnV, AV, K_232_, and K_268_ coefficients, and the TOTOX indexes, which collectively describe the formation of primary and secondary oxidation products and the accumulation of free fatty acids during accelerated storage.

At the beginning of storage, all oxidation parameters of fresh, cold-pressed rapeseed oil (O) exhibited the lowest values of the evaluated quality parameters (PV = 0.50 meq O_2_/kg, pAnV = 0.56, TOTOX = 1.56, AV = 0.09 mg NaOH/g, K_232_ = 1.101, and K_268_ = 0.084), but these values increased progressively with storage time (Figure 2). According to Codex Alimentarius [30], acceptable limits are PV ≤ 15 meq O_2_/kg, AV < 4.0 mg KOH/g oil, and pAnV < 10. Almost none of the analyzed samples exceeded these limits, indicating that, based on these indices, the oils could be stored longer than typically recommended by manufacturers.

Monitoring oxidation products is crucial because they not only affect oil colour, flavour, and odour but also have adverse effects on health. PV is an indicator of the formation of primary oxidation products, mainly hydroxyperoxides, whereas pAnV reflects the degree of secondary oxidation products. It is noteworthy that the primary oxidation product is unstable and, rapidly transforms to secondary products. The TOTOX index, through combining both primary and secondary oxidation products, gives information about the overall oil quality. AV provides information on hydrolytic degradation by measuring the amounts of free fatty acids, which accelerate the oxidation process. Additionally, the specific extinction coefficients K_232_ and K_268_ provide information about structural changes in unsaturated fatty acids. Therefore, analyzing all these indicators is crucial to a comprehensive assessment of oil quality [7].

A pronounced increase in PV was observed during the first stage of the experiment, from 0.50 meq O_2_/kg initially for fresh cold-pressed rapeseed oil (O) to 3.75–5.32 meq O_2_/kg for oils after the first week of storage. Meanwhile, oils stored under accelerated conditions for 2 weeks had similar PV results, ranging from 3.64 to 5.59 meq O_2_/kg. The Duncan test revealed no significant differences between samples after one and two weeks of storage, except for oil stored in Dorica dark glass bottles. A slight reduction in the amount of hydroperoxides was observed in oil samples containing GPE film strips in clear glass bottles, likely resulting from the decomposition of primary oxidation products into volatile and non-volatile secondary compounds such as aldehydes and ketones. This behaviour may be associated with the light-induced reactivity of ethyl sinapate. Interestingly, previous studies on hydroxycinnamic acid alkyl esters in refined rapeseed oil did not report such effects [31,32].

As expected, PVs were higher in oil samples stored in clear glass bottles (4.58–5.32 meq O_2_/kg and 5.02–5.59 meq O_2_/kg after 7 and 14 days, respectively) compared to those in dark glass bottles (3.76–4.44 meq O_2_/kg and 4.67–5.06 meq O_2_/kg, after 7 and 14 days, respectively). The addition of GPE film strips to dark glass bottles did not affect peroxide formation in the first week, but a slight inhibition was observed during prolonged storage, particularly in Marasca bottles. Conversely, adding film strips to clear glass bottles inhibited peroxide formation in Dorica bottles, whereas oil samples stored in Marasca bottles exhibited a slightly higher PV after the first week and a lower one after the second.

The shape of the bottle is not only an aesthetic feature but also influences oxidation kinetics [33]. In addition to glass colour, the oxygen concentration in the headspace plays a major role in oxidation [3,33]. The Dorica bottle, being taller and more cylindrical, has a smaller surface area-to-volume ratio compared to the flatter, square Marasca bottle. Theoretically, this geometry should enhance oxidative stability by minimizing oil–wall contact, reducing headspace, and limiting light and heat exposure. However, the obtained results did not support these assumptions, as they showed similar or slightly higher contents of peroxides in oils stored in Dorica bottles (Duncan test).

The pAnV increased significantly in all tested samples during storage (pAnV = 0.85–1.22 after 1 week; 1.08–2.27 after 2 weeks). The highest rise in secondary oxidation products was observed in clear glass bottles without film strips, where pAnV approximately doubled each week (0.56, 1.17–1.22, and 2.06–2.27 for fresh oil and after the 1st and 2nd week of storage, respectively). As expected, in dark bottles where the autoxidation process should be dominated by limiting light access and thus inhibiting photooxidation, the formation of secondary oxidation products was smaller but still evident, particularly in the second week (pAnV = 0.85–1.22 and 1.36–1.53 after the 1st and 2nd week of storage, respectively).

The inclusion of GPE film strips effectively suppressed the formation of secondary oxidation products, especially in clear glass bottles. This effect is likely due to the diffusion of ethyl sinapate from the film into the oil, where it acts as an antioxidant, potentially synergistically interacting with native oil compounds. Additionally, the significantly protective effect of the cylindrical Dorica bottle shape used was not observed. These results were quite unexpected, even if they had already been observed in the case of PVs.

The lower PV and pAnV values observed in oils containing GPE film strips confirm the antioxidative potential of the active packaging material. The combined oxidation index (TOTOX) integrates both PV and pAnV, providing a comprehensive measure of oxidative deterioration. According to the German Guidelines for Edible Fats and Oils [34], a TOTOX value below 20 indicates good oil quality, while some studies suggest that 10 should be the limit for high-quality oils. None of the analyzed oil samples exceeded 20 of the TOTOX index. However, TOTOX indicators exceeded 10 for some oils stored for one week (generally, samples in clear glass and Marasca bottles). After two weeks, only oils with film strips stored in Dorica clear glass bottles and Marasca dark glass bottles maintained TOTOX values below this limit.

The extinction coefficients K_232_ and K_268_ are related to conjugated dienes (primary oxidation products) and conjugated trienes (secondary oxidation products), respectively. Currently, there are no regulations in the European Union, the Codex Alimentarius, or the FDA regarding the K_232_ and K_268_ limits for cold-pressed oils. These parameters are specified only for olive oil. Despite the lack of a specific value for these indicators, they are a quick and simple source of information about the oxidative deterioration of oil.

The initial K_232_ and K_268_ values for fresh cold-pressed rapeseed oil (O) were 1.101 and 0.084 a.u., respectively. Similar to PV and pAnV results, both extinction coefficients increased over time. The highest values were observed for samples stored in clear glass bottles without film strips (K_232_ = 1.762–1.828 and K_268_ = 0.154–0.223 after the first week, as well as K_232_ = 1.781–1.813 and K_268_ = 0.247–0.263 after the second week). The lowest values were recorded for oils stored in dark glass bottles containing GPE film strips (K_232_ = 1.236–1.428 and K_268_ = 0.102–0.154 after the first week, as well as K_232_ = 1.555–1.558 and K_268_ = 0.185–0.202 after the second week).

Another important factor related to the durability and quality of oils is the AV. It is well known that the lower the AV, the more stable the oil. According to the Codex Alimentarius, the AV should not exceed 4 mg KOH/g oil. It is worth noting that the fresh cold-pressed rapeseed oil (O) had the lowest initial AV (0.09 mg KOH/g). During the accelerated storage period, the formation of free fatty acids increased, reaching AVs between 0.58–1.13 mg KOH/g oil after the first week and 0.63–1.83 mg KOH/g oil after the second week. It is essential to emphasize that throughout all experiments, AV remained below the threshold limit, regardless of bottle colour or the inclusion of the film strips. As observed, only samples stored in dark glass Dorica bottles, both with and without the film addition, showed no statistically significant increase in AV (Duncan test). As expected, and consistent with previous findings, storage in dark glass containers resulted in a slower oxidation rate, as evidenced by the significantly lower formation of free fatty acids. Interestingly, the incorporation of the gelatin/polyvinyl alcohol film strips containing ethyl sinapate did not significantly affect the AV of the analyzed oil, except for oils stored in Marasca bottles. The first week, slightly higher AVs were observed for oils stored in dark glass bottles without film incorporation, whereas the opposite trend was noted in the second week. Therefore, no clear protective or pro-oxidative effect can be confirmed.

For comparison, differences in the increase in oxidative parameters (PV, pAnV, acidity, K_232_, K_268,_ and TOTOX) during storage of vegetable and extra virgin olive oils in bottles of different glass colours have been widely studied [4,24,35,36].

The PV as an indicator of the initial oxidation stage demonstrated significant differences between oils stored in clear and dark glass bottles. Kishimoto et al. [37] reported that after 1 week of sunlight exposure at room temperature, olive oil stored in dark glass reached about 40 meq O_2_/kg, while oil in clear glass reached approximately 80 meq O_2_/kg. After 5 weeks, these values increased to approximately 170 meq O_2_/kg and 300 meq O_2_/kg, respectively. It should be noted that even though dark glass provided better protection, PV still exceeded the legal limit for olive oil (20 meq O_2_/kg). Similarly, Guil-Guerrero and Urda-Romacho [36] observed an increase in PV across three olive oil varieties stored in both clear and dark glass. However, when bottles were flushed with nitrogen and stored at 20 °C in the dark, the increase in PV after 12 months was much lower (Picul variety: +5.9 vs. +5.6 meq O_2_/kg; Hojiblanca variety: +9.4 vs. +5.3 meq O_2_/kg; Arbequina variety: +9.1 vs. +4.3 meq O_2_/kg for clear and dark glass, respectively). In another study, the impact of storage conditions (temperature, glass colour, light exposure, and bottle headspace) on vitamin D_3_ degradation in fortified rapeseed oil was examined. Although pAnV remained below 10 in all cases, PV strongly depended on storage conditions, with values several times higher for oils exposed to sunlight and air. Additionally, the authors linked vitamin D_3_ degradation directly to oil oxidation [35]. The extinction coefficient K_270_, correlated with carbonyl compounds and secondary oxidation products, also increased progressively in transparent glass, while remaining lower in amber glass. Nevertheless, even in dark glass, K_270_ exceeded the acceptable limit after 3 weeks of storage, confirming the need for additional protective strategies. Notably, amber glass effectively inhibited triacylglycerol degradation (FFA formation), in contrast to clear glass—consistent with our findings [37]. Conversely, Guil-Guerrero and Urda-Romacho [36] observed no significant differences in K_270_ or FFA after one year, regardless of bottle colour, with the largest difference being only 0.02% for the Arbequina variety.

The use of dark glass primarily reduces oil autoxidation but is often insufficient on its own. Consequently, numerous studies have focused on developing active packaging systems incorporating antioxidants to prolong oil quality. Similar to our findings, the incorporation of antioxidants into polymer matrices enables the gradual migration of active compounds, enhancing oxidative stability. In these studies, oils were either packed in heat-sealed sachets or—similarly to the present work—films were directly immersed in oil [38,39,40,41].

Across these studies, regardless of the antioxidant type or oil used, inhibition of both primary and secondary oxidation products was consistently observed. For instance, starch-based films with encapsulated eugenol (S–EOA–L) effectively slowed the formation of primary oxidation products (PV, K_232_) compared to oil packed in low-density polyethylene (PE) sachets or Petri dishes. After 18 days, PVs were 9.4, 135.8, and 115.8 meq O_2_/kg for oils stored in corn starch films, open dishes, and PE, respectively, while oil stored with S–EOA–L films reached only ~3 meq O_2_/kg. These high PVs for unprotected samples were attributed to high oxygen availability. However, no significant differences were observed in K_268_, indicating that secondary oxidation had not yet progressed [38]. Stoll et al. [40] conducted complementary research using poly(lactic acid) (PLA) films with carotenoid extracts for sunflower oil preservation. The experiment included direct addition of extract, immersion of films in oil, and oil storage in sachets under light and dark conditions. Carotenoids such as β-carotene and lycopene may act as pro-oxidants at high concentrations, whereas their incorporation into polymer films and controlled release results in antioxidative behaviour. Moreover, carotenoid-enriched films enhanced both oxygen and light barrier properties of the packaging.

It should also be emphasized that not only the colour of glass bottles but also the lipophilization of natural antioxidants significantly improves oil protection. Similar to our approach, Arslan et al. [6,8] demonstrated that alkyl esters of sinapic acid exhibit higher antioxidative efficiency in lipophilic environments. Microencapsulation of flaxseed oil with palmitoyl or hexyl sinapate effectively inhibited oxidation progression. In particular, palmitoyl sinapate reduced PV increases by nearly half compared to unprotected oil. Moreover, K_232_ was 3.5-fold lower compared to bulk oil and nearly 2-fold lower than for oil encapsulated with hexyl sinapate. The inhibition of secondary oxidation (K_270_) was less pronounced, consistent with our findings. The enhanced solubility of nonpolar antioxidants and their ability to form a protective barrier around oil droplets explain this effect [6]. Therefore, combining lipophilic antioxidant-loaded films with dark glass packaging offering high oxygen and light barrier properties appears to be an effective strategy to prevent rancidity and preserve oil quality during storage.

### 3.2. Changes in Antioxidant Properties of Cold-Pressed Rapeseed Oils During Accelerated Storage

**Figure 3:**
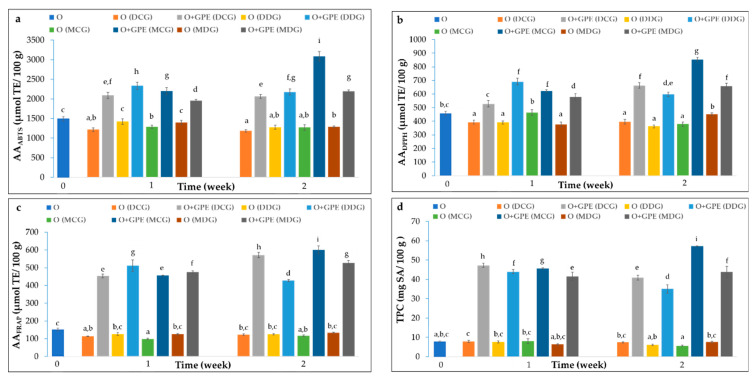
Changes in ABTS (**a**), DPPH (**b**), FRAP (**c**), and TPC (**d**) results of cold-pressed rapeseed oils (O) without and with gelatin/polyvinyl alcohol film strips loaded with ethyl sinapate (GPE) stored for two weeks under accelerated conditions in Dorica clear glass bottles (DCG), Dorica dark glass bottles (DDG), Marasca clear glass bottles (MCG), and Marasca dark glass bottles (MDG). Bars with different letters indicate significant differences (one-way ANOVA and Duncan test, *p* < 0.05).

The development of innovative materials capable of releasing antioxidants into food provides tangible benefits for food preservation. The presence of active compounds with radical scavenging properties is crucial for preventing oxidative deterioration and quality loss caused by free radicals. The results of AA and TPC of cold-pressed rapeseed oils stored under accelerated conditions in different types of bottles, with or without immersion of the GPE film strips, after 7 and 14 days, are summarized in Figure 3.

The obtained AA and TPC results determined using various analytical methods differ significantly from each other. These discrepancies arise from the underlying reaction mechanisms of the applied assays and, consequently, from the specific classes of bioactive compounds each method can detect. The DPPH and ABTS assays are both based on free radical scavenging mechanisms, which are particularly relevant for assessing oil protection strategies against oxidation. Naturally occurring antioxidants in cold-pressed oils—primarily phenolic compounds, tocopherols, carotenoids, and sterols—can neutralize free radicals responsible for initiating lipid oxidation [42].

As can be seen, ABTS values in all tested samples were significantly higher—up to five times—than those obtained from the DPPH assay (Figure 3a,b). The ABTS method primarily follows a single electron transfer (SET) mechanism; however, under certain conditions, such as with “slow-reacting” antioxidants or those with steric hindrance, it may also involve hydrogen atom transfer (HAT), leading to a mixed SET–HAT mechanism [43]. Conversely, the DPPH assay operates through a mixed mechanism involving SET, HAT, and proton-coupled electron transfer (PCET) processes, with the dominant pathway depending on reaction conditions. Moreover, ABTS can detect both hydrophilic and hydrophobic antioxidants, while DPPH, due to its hydrophobic nature, is more selective toward lipophilic antioxidants. The third applied method, FRAP, also relies on a SET mechanism. This colorimetric assay measures the antioxidant’s reducing ability by converting the colourless Fe^3+^–TPTZ (2,4,6-tripyridyl-s-triazine) complex into the intensely blue Fe^2+^–TPTZ complex [44]. The TPC, determined using the F–C method, is widely applied in oil analysis due to its capacity to quantify total phenolic compounds regardless of structural diversity. However, this analytical test is prone to interference from various compounds, including oxidized polyphenols, since it provides only an approximate overview of total polyphenol content. The proposed AA and F–C methods were conducted at various pH levels, FRAP assay under acidic conditions (pH 3.6), F–C assay under alkaline conditions, which enable the phenolic hydroxyl groups to deprotonate, forming phenolate anions that can effectively reduce the F–C reagent, while ABTS and DPPH tests do not have a specific pH [42].

It is important to note that ethyl sinapate, a phenolic acid ester derived from sinapic acid, significantly increases the TPC value upon its release from the film strips into the oil (Figure 3d). The AA results demonstrate that ethyl sinapate is an efficient electron donor, exhibiting enhanced AA in all applied assays regardless of the reaction medium (Figure 3a–c). As a phenolic compound, it can undergo both SET and HAT reactions, supported by strong resonance stabilization of the resulting phenoxyl radicals and additional metal-chelating capacity. In lipid systems, the HAT mechanism is fundamental because phenolics inhibit oxidation by donating a hydrogen atom to alkylperoxyl radicals (ROO^•^) formed during the initiation phase of lipid oxidation [42].

Moreover, interactions between released ethyl sinapate and endogenous oil components, such as tocopherols, phospholipids, or minor phenolics naturally present in cold-pressed rapeseed oil, may modulate its measurable activity, either enhancing or attenuating it. These interactions become more pronounced over time, contributing to the observed variation among analytical methods [20].

It can be noted that the initial AA values of fresh cold-pressed rapeseed oil (O), determined by ABTS (1433.66 µmol TE/100 g), DPPH (479.95 µmol TE/100 g), and FRAP (142.61 µmol TE/100 g) assays, were higher than the AA of oil samples stored without the addition of immersed film strips (Figure 3a–c). It is well established that antioxidants naturally present in oils gradually degrade during storage, leading to a diminished protective effect against oxidation. This process is one of the main motivations for incorporating antioxidants directly into oils or into packaging materials to enhance their oxidative stability. Interestingly, a slightly higher TPC value was observed in cold-pressed rapeseed oil samples stored without film strips during the 7-day storage period, although the difference was not statistically significant (Figure 3d, Duncan test). Only oil stored in MCG bottles showed a significant decrease in TPC compared to fresh oil. Overall, the results confirmed a decrease in AA and a slight reduction in TPC with increasing storage time. As presented in Figure 3, the highest degradation rates were observed for oils stored in clear glass bottles—specifically, Dorica bottles analyzed by the ABTS assay (17% decrease) and Marasca bottles analyzed by the FRAP assay (31%) and TPC (22%). Surprisingly, only slight, statistically insignificant differences between the AA of oils stored in clear and dark glass were detected by the DPPH method. These results confirm a modest protective effect of dark glass bottles against photooxidation induced by light-sensitive components in the oil. The Duncan test also indicated that the differences in AA and TPC were independent of bottle geometry. The effect of bottle shape would likely become more pronounced with a lower filling level (larger headspace), as the air–oil contact area is greater in square Marasca bottles than in cylindrical Dorica bottles. Despite the absence of significant differences between glass types in DPPH results, this assay revealed the highest decrease in radical scavenging activity compared to fresh oil (15–24%), followed by FRAP (6–19%) and ABTS (10–17%) after 14 days of accelerated storage. Because the DPPH method is more responsive to lipophilic antioxidants, these findings suggest that hydrophobic antioxidants are consumed more rapidly during the oxidation process. An interesting trend, distinct from that observed with the ABTS or DPPH results, was noted during the TPC analysis. It is known that phenolic compounds are sensitive to light exposure, which accelerates the oxidation of oil. Therefore, the oil storage in transparent glass increases the photodegradation of oil, due to better access to UV light compared to a dark glass bottle [24]. Rover and Brown [45] confirmed the disadvantage of F–C methods due to their nonspecific reaction. The F–C reagent can also react with non-phenolic compounds, including “easily oxidizable molecules”. Besides phenolics, the F–C reagent can be reduced by aromatic amines, sulphur dioxide, ascorbic acid, other endiol-containing compounds, and proteins [44]. These limitations lead to the selection of various analytical methods based on different mechanisms to determine antioxidant properties of oils.

Generally, the changes observed in primary and secondary oxidation products correspond with the amount of antioxidant substances in oil samples. Evidently, oil samples packaged in various glass bottles with GPE film strips had higher AA and lower amounts of oxidation products. This suggests that ethyl sinapate released into the oil effectively inhibited oxidation processes by both scavenging free radicals and effectively reducing pro-oxidants. Another interesting observation is that oils in clear glass bottles exhibited higher AA and higher contents of both primary and secondary oxidation products. This is likely due to both photooxidative processes initiated by UV light and the increased rate of ethyl sinapate release into the oil, also resulting from the presence of light. It appears that both processes compete with each other.

The degradation of antioxidants during storage inevitably limits their protective effect. Therefore, supplementing oils with antioxidants, either directly or via active packaging, is a promising strategy to extend shelf life. Recent studies have demonstrated that the controlled release of antioxidants from packaging materials provides superior protection compared to direct supplementation [40,41]. In the present study, a gradual migration of ethyl sinapate from the GPE film into the oil was observed, resulting in a sustained antioxidant effect. Immersion of the GPE film strips caused approximately a 1.4–1.7-, 1.3–1.8-, 3.8–4.6-, and 5.7–6.5-fold increase in the AA and TPC results compared with oil samples without film strips, as determined by the ABTS, DPPH, FRAP, and TPC methods, respectively (Figure 3). After 14 days of storage, the enhancement effect of the antioxidant present in the added film strips to the studied oils further increased to 1.7–2.4-, 1.6–2.3-, 3.4–5.2-, and 5.5–10.0-fold, respectively, relative to the control samples. Moreover, AA measured by both DPPH and FRAP assays increased by approximately 14–37% and 11–31%, respectively, after 14 days compared to 7 days, except for oil stored in Dorica dark glass bottle. Interestingly, greater improvements in the AA were observed for oils stored in Marasca clear glass bottles compared to those stored in dark glass bottles, as indicated by all applied assays. This phenomenon may be attributed to the enhanced release of ethyl sinapate from the polymer matrix due to easier access to light. Esterification of phenolic acids, such as sinapic acid, introduces an alkyl chain, increasing molecular mass and lipophilicity. These changes slow down the migration of the compound from the polymer into the food matrix, resulting in a more controlled diffusion and prolonged antioxidant protection. As reported by Almasi et al. [41], the migration process depends on diffusion, dissolution, and equilibrium dynamics. Therefore, the incorporation of ethyl sinapate into the polymeric film not only increased its lipophilicity but also improved its functional stability, positively influencing the oxidative protection of the stored oil. Therefore, esterification increased not only the lipophilicity but also the molecular weight of the compound, which in turn enhanced the protective properties of the resulting films.

Many studies have demonstrated a decrease in AA and TPC results to varying degrees, depending on storage conditions and packaging types, as storage time increases [4,31,37]. For comparison, olive oil stored in clear glass bottles showed a significantly greater reduction in phenolic content (from approximately 600 mg/kg to 300 mg/kg) than oil stored in dark glass (from 600 mg/kg to 50 mg/kg) [37]. It is noteworthy that the most pronounced decline occurred during the first week of storage, while the parameter remained nearly constant between weeks 3 and 5. This rapid initial consumption of antioxidants confirms the necessity of a controlled and sustained release of active compounds to extend the oil’s shelf life. In agreement with the presented results, enhanced DPPH radical scavenging activity was also observed in soybean oil packaged in PLA films incorporated with tert-butylhydroquinone (TBHQ) [41]. These authors emphasized that the composition of the film plays a crucial role: increased porosity accelerates the release of antioxidants, potentially causing the protective effect of the packaging to diminish prematurely.

This relationship is further supported by studies on hexyl and palmitoyl sinapate, where microencapsulated flaxseed oil enriched with sinapic acid esters and stored in sealed dark glass containers exhibited a continuous decrease in DPPH radical scavenging activity and TPC over 45 days at 24 °C [6]. Nevertheless, hydroxycinnamic acid esters demonstrated a significant protective effect, increasing the AA and TPC of flaxseed oil by approximately 2–3-fold. These findings are consistent with the broader research trend, indicating that hydroxycinnamic acid esters can act as effective antioxidants, enhancing the oxidative stability and quality of vegetable oils.

### 3.3. Synchronous Spectra of Cold-Pressed Rapeseed Oils During Accelerated Storage

**Figure 4:**
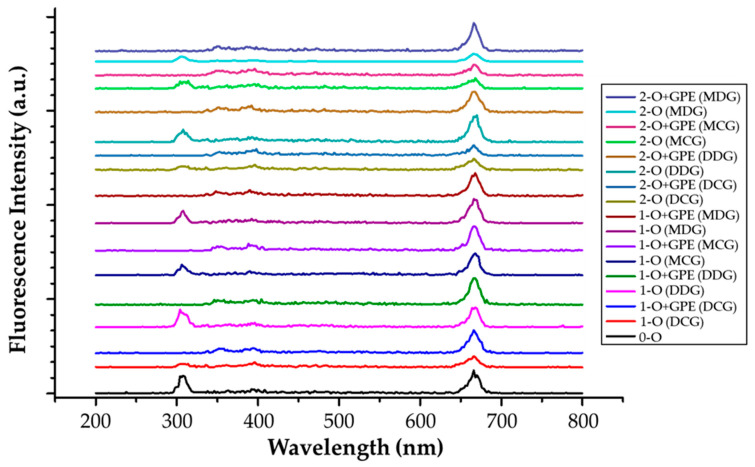
Synchronous fluorescence spectra of cold-pressed rapeseed oils (O) without and with gelatin/polyvinyl alcohol film strips loaded with ethyl sinapate (GPE) diluted in n-hexane and recorded at Δλ = 10 nm during accelerated storage test.

Figure 4 displays the synchronous fluorescence spectra recorded for cold-pressed rapeseed oils packed in different types of bottles, both without and with GPE film strips, during accelerated storage. The applied excitation–emission offset (Δλ = 10 nm) was selected based on our previous research [7], as it provided optimal visibility of spectral features and fluorescence intensities for tocopherols, chlorophylls, and oxidation products.

As shown in Figure 4, the fluorescence profiles of cold-pressed rapeseed oil samples varied noticeably depending on the storage conditions. Three main regions of interest can be distinguished in the obtained spectra, corresponding to specific excitation ranges. The maximum band observed at approximately 310 nm is attributed to tocopherols, and it also falls within the characteristic absorption range of polyphenolic compounds (250–400 nm). The bands between 640–680 nm are associated with chlorophylls a and b, as well as their degradation products, pheophytins a and b. Furthermore, SF spectroscopy enables monitoring of the oxidation process by tracking the emergence of bands assigned to conjugated hydroperoxides in the range of 440–470 nm [46]. The storage process induced changes in the fluorescence intensities, including shifts, disappearance, or appearance of bands. Therefore, SF spectroscopy is described as a rapid, cost-effective, and non-destructive method for monitoring the quality of edible oils. Nevertheless, even with the selected interval, some spectral overlap between different fluorophores naturally present in edible oils was observed.

It is evident from Figure 4 that the fluorescence band corresponding to tocopherols exhibited higher intensity in oils stored in dark glass bottles compared to those stored in clear glass bottles. Similar to our previous observations, the addition of film strips containing sinapic acid ester resulted in both a decrease in intensity and a slight shift in the tocopherol-associated band from 310 to 340 nm. Furthermore, the spectra of oils enriched with GPE film strips and stored in both Marasca and Dorica clear glass bottles demonstrated an apparent decrease in the chlorophyll band (λmax = 668 nm) after seven days of accelerated storage. It can also be noted that the fluorescence maxima associated with tocopherols and chlorophylls gradually decreased during storage, indicating oxidative degradation of these compounds. This decline was less pronounced in oils stored in dark glass bottles, confirming their superior protection against photooxidation compared to oils stored in clear glass bottles. Similar conclusions regarding the applicability of fluorescence spectroscopy for tracking oxidative changes in edible oils, particularly during heating, have been reported in recent studies [47]. Moreover, SF spectroscopy enables the observation of new fluorophores formed as a result of antioxidant addition or oil blending, providing valuable insights into compositional and oxidative changes [46,47,48].

### 3.4. Chemometric Analysis

Multivariate data analysis was performed to discriminate the quality of oil samples stored in different types of bottles without and with GPE film strips by applying principal component analysis (PCA) and hierarchical clustering analysis (HCA), representing both sub-classes visualization and agglomerative algorithms, respectively.

#### 3.4.1. Principal Component Analysis

**Figure 5:**
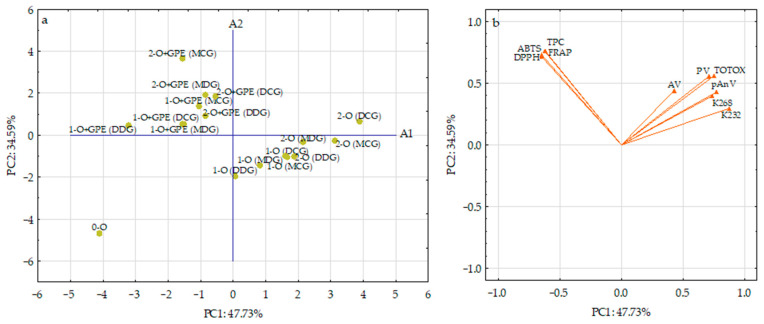
Principal component analysis plots. (**a**) PCA scores plot for the studied cold-pressed rapeseed oils (O) without and with gelatin/polyvinyl alcohol film strips loaded with ethyl sinapate (GPE) and stored for 1 and 2 weeks in different bottle types (DCG—Dorica clear glass, DDG—Dorica dark glass, MCG—Marasca clear glass, MDG—Marasca dark glass) under accelerated conditions; (**b**) loading plots for different variables on PC1 and PC2.

Oxidative status and antioxidant properties of cold-pressed rapeseed oil samples stored during two weeks in various bottle types without and with GPE film strips were analyzed using PCA on ten different variabilities (PV, pAnV, TOTOX, AV, K_232_, K_268_, ABTS, DPPH, FRAP, and TPC) to obtain information about the interrelationship among variables and grouping of oils on a similarity basis. Among the nine components, PC1 and PC2 revealed eigenvalues higher than 1 (4.773 and 3.459), accounting for 47.73% and 34.59% of the variance, respectively. Together, these two components explain 82.32% of the total variance, capturing the majority of the information within the dataset. However, the remaining seven principal components (PC3, PC4, PC5, PC6, PC7, PC8, and PC9) had eigenvalues progressively lower than 1 (0.755, 0.497, 0.294, 0.122, 0.0749, 0.0155, and 0.00894, respectively) and did not explain the variability in the data (<17.68% total). Therefore, according to Kaiser’s rule [49], only the first two PCs were used for further study. The results of PCA calculations were plotted using a two-dimensional score scatterplot (Figure 5a). However, the relationships between the first two principal components and the studied variables were presented graphically by the loading plot in Figure 5b.

The PC1 was positively correlated (0.4271–0.8744) with oxidative parameters of the investigated oils (PV, pAnV, TOTOX, K_232_, and K_268_), while the inverse correlations were observed between PC1 and their antioxidant features determined by ABTS (−0.6488), DPPH (−0.6487), FRAP (−0.6236) assays and TPC analyzed by F-C test (−0.6206). Meanwhile, PC2 highly positively contributed to the antioxidant potential of oil samples analyzed by all analytical assays (0.7162−0.7640), as well as amounts of primary oxidative products (PV) (0.5583) and total oxidative status (TOTOX) (0.5602).

The score plot of PCA of the first two components showed a clear and distinct separation of the investigated oils (Figure 5a). A group of oils, packed and stored in different glass bottle types with dipped GPE film strips, having high antioxidant properties (ABTS = 1956.78–3086.47 µmol TE/100 g, DPPH = 528.29–854.37 µmol TE/100 g, FRAP = 428.00–599.76 µmol TE/100 g, and TPC = 35.02–57.19 mg SA/100 g) and low oxidative parameters (PV = 3.64–5.11 meq O_2_/kg, pAnV = 1.03–1.61, TOTOX = 8.53–11.39, AV = 0.63–1.83 mg NaOH/g, K_232_ = 1.236–1.675, and K_268_ = 0.102–0.237), was located on the left half of the plot and had negative values for PC1 and positive values for PC2. However, oils without GPE film strips, stored in the same bottles under identical accelerated conditions, created an evidently distinct group on the right-hand side of Figure 5a, and had positive values for PC1. These oil samples contained somewhat higher amounts of primary (PV = 3.76–5.59 meq O_2_/kg, K_232_ = 1.452–1.828) and secondary (pAnV = 0.85–2.27, K_268_ = 0.154–0.263) oxidation products, free fatty acids (AV = 0.67–1.82 mg NaOH/g), and total oxidation index (TOTOX = 8.74–13.45), while their antioxidant features (ABTS = 1184.20–1422.80 µmol TE/100 g, DPPH = 362.62–464.13 µmol TE/100 g, FRAP = 98.28–133.75 µmol TE/100 g, and TPC = 5.71–8.02 mg SA/100 g) were significantly lower compared to samples fortified with GPE film strips. Evidently, the location of oil stored 2 weeks in a Dorica clear glass bottle (2-O (DCG)) in the higher right-hand quadrant of Figure 5a (positive values for PC1 and PC2) indicates its highest oxidative parameters (all oxidative parameters were situated in this region of the PCA plot, Figure 5b). This plotting segregation of the studied oil samples can be explained by the fact that ethyl sinapate released from GPE film strips had a good antioxidant potential and inhibitory activity against the generation of primary and secondary oxidation products in oil samples. Interestingly, fresh cold-pressed rapeseed oil (0-O) with the lowest oxidative parameters (PV = 0.50 meq O_2_/kg, pAnV = 0.56, TOTOX = 1.56, AV = 0.09 mg NaOH/g, K_232_ = 1.101, and K_268_ = 0.084) and moderate antioxidant capacity (ABTS = 1433.66 µmol TE/100 g, DPPH = 479.95 µmol TE/100 g, FRAP = 142.61 µmol TE/100 g), and total phenolic amount (TPC = 7.29 mg SA/100 g) was situated some distance away from all of the other oil samples (the lower left-hand quadrant of Figure 5a). This indicates that its composition regarding antioxidant parameters and oxidative products differed significantly from the oil samples packed in various bottle types and treated with accelerated storage periods.

The PCA conducted in this work confirmed that ethyl sinapate, present in GPE film strips added to bottles, played a significant role in enhancing oil quality stored during the accelerated storage. Moreover, this unsupervised method indicated some common bioactive properties and oxidative status of the stored oils, owing to which it was possible to divide them into characteristic groups.

#### 3.4.2. Hierarchical Cluster Analysis

In this study, HCA was also performed to classify the oil samples in clusters in terms of their nearness or similarity based on the investigated variables, including oxidative parameters and antioxidant properties. Additionally, HCA was applied to reveal relationships between the antioxidant potential analyzed by three different analytical methods (ABTS, DPPH, FRAP), amounts of TPC and oxidative parameters (PV, pAnV, TOTOX, AV, K_232_, and K_268_) of the studied oils stored in various bottles without and with antioxidant agents during 2 weeks under accelerated conditions.

**Figure 6:**
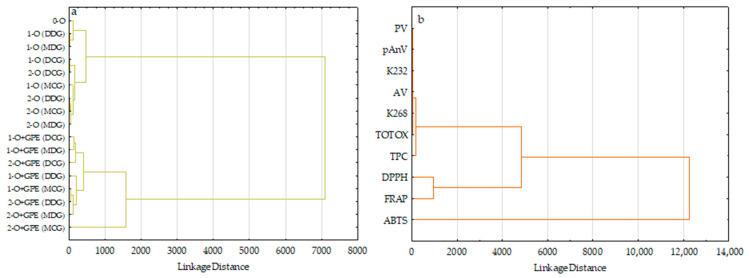
Dendrograms visualizing the hierarchical clustering of (**a**) the studied cold-pressed rapeseed oils (O) without and with gelatin/polyvinyl alcohol film strips loaded with ethyl sinapate (GPE) and stored for 1 and 2 weeks in different bottle types (DCG—Dorica clear glass, DDG—Dorica dark glass, MCG—Marasca clear glass, MDG—Marasca dark glass) under accelerated conditions, and (**b**) their oxidative and antioxidative parameters.

HCA results were depicted as dendrograms (Figure 6), in which two well-defined clusters of investigated oils (Figure 6a) and two clusters of variable sets (Figure 6b) were presented.

Similarly to the results found by PCA, the dendrogram depicted in Figure 6a shows clear separations of oils stored in various bottles without film strips, which possessed low antioxidant potential and oxidative stability from the second group, including oils packed in the same bottles containing antioxidant film strips, because these samples had high antioxidant characteristics and low oxidative contents of oxidation products. Nevertheless, both clusters included two inter-clusters consisting of oils after the 1st and 2nd week of storage, respectively. This can be explained by the fact that cold-pressed rapeseed oil is susceptible to oxidation reactions, especially during more extended storage period, while ethyl sinapate, presented in film strips dipped in oils, had an essential role in inhibiting these destructive reactions, enhancing antioxidant properties and reducing the generation of oxidation products. Interestingly, oil stored 2 weeks in a Marasca clear glass bottle after dropping antioxidant film strips (2-O+GPE (MCG)) with the highest antioxidant capacity analyzed by three analytical methods (ABTS = 3086.47 µmol TE/100 g, DPPH = 854.37 µmol TE/100 g, and FRAP = 599.76 µmol TE/100 g), the total amount of phenolics (TPC = 57.19 mg SA/100 g) lay at some distance from the second cluster.

As shown in Figure 6b, the first cluster was divided into two subgroups. The first subgroup encompassed all determined oxidative parameters and TPC in the studied oils, whereas the second subgroup was composed of DPPH and FRAP methods. However, the second cluster was created solely by the ABTS test. This suggests that the phenolic compounds analyzed by F–C assay can more effectively inhibit oxidative processes in cold-pressed rapeseed oils during accelerated storage, evaluated by the determined parameters, than other hydrophobic and hydrophilic antioxidants able to scavenge DPPH radicals or ABTS cation radicals, as well as the reduction of (Fe(III)-TPTZ) to an intense blue colour (Fe(II)-TPTZ) complex.

The results confirm that HCA can help classify oil samples based on their antioxidant features and oxidation parameters.

#### 3.4.3. Pearson Correlation Analysis

**Figure 7:**
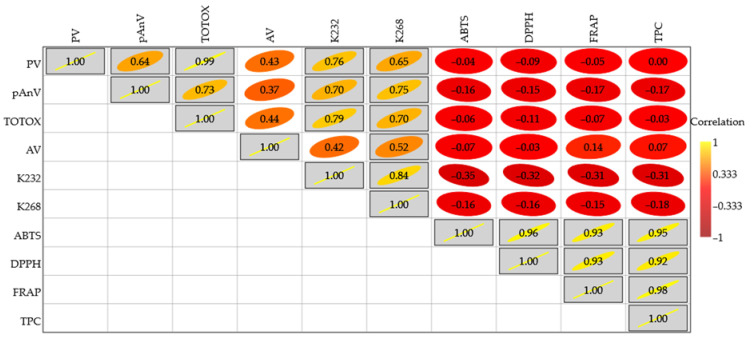
The heatmap of correlations between oxidation parameters and antioxidant properties of the studied cold-pressed rapeseed oils without and with gelatin/polyvinyl alcohol film strips loaded with ethyl sinapate and stored for 1 and 2 weeks in different bottle types under accelerated conditions (grey boxes indicating significant relationship, *p* < 0.05).

Pearson correlation analysis was performed, and the calculated correlation coefficients between oxidation parameters and antioxidant properties of the investigated oil samples were illustrated as a correlation heatmap (Figure 7).

It is noteworthy that there were strong and positive correlations (r = 0.64–0.99) between the TOTOX indexes and contents of primary and secondary oxidation products generated in the studied oils and determined as PV and pAnV, respectively. Obviously, increasing concentrations of oxidative products caused the increase in the oxidation state given by the TOTOX values. Furthermore, significant positive relationships were found between conjugated dienes (K_232_) and trienes (K_268_) (r = 0.84), and conjugated polyenes and concentrations of primary (PV) and secondary (pAnV) oxidation products, as well as TOTOX values (r = 0.65–0.79). These positive relations between PV and K_232_, as well as pAnV and K_268_, indicate that the absorbances at 232 nm and 268 nm for the investigated oil solutions in hexane generally reflect the amounts of primary oxidation products (conjugated peroxides), and secondary oxidation products (conjugated trienes). Similarly, significant and positive correlations were observed among AA results of all studied oils determined using three analytical methods: ABTS, DPPH, and FRAP (r = 0.93–0.96). This suggests that antioxidants present in the tested oils could scavenge ABTS and DPPH radicals and create an intense blue ferrous-tripyridyltriazine complex (Fe(II)-TPTZ) at the same time. Additionally, phenolic compounds measured by F–C assay in these oils contributed significantly to their antioxidant potential. Therefore, calculated Pearson correlation coefficients between TPC and ABTS, DPPH, and FRAP results varied from 0.92 to 0.98 (Figure 7). In contrast, free fatty acids measured as AV insignificantly positively affected the contents of peroxides (PV) and conjugated polyenes (K_232_ and K_268_) (r = 0.37–0.44). Unexpectedly, the heatmap illustrated the lack of associations between antioxidant features determined by chosen analytical methods and the oxidative status of the oil samples stored in different bottles during two weeks at accelerated conditions. These results confirmed that the contents of oxidative products in the investigated oils were not affected by the antioxidant potential evaluated by the selected analytical methods.

## 4. Conclusions

The prepared gelatin/polyvinyl alcohol film strips, incorporating ethyl sinapate, effectively protected cold-pressed rapeseed oil against oxidation during accelerated storage test. The application of GPE film strips reduced primary oxidation products by 1–35% and secondary oxidation products by 13–41% in the stored oils, depending on bottle shape and colour, compared to oil samples packed without active film. These findings suggest that ethyl sinapate efficiently inhibited both photooxidation and autoxidation processes in cold-pressed rapeseed oil. During storage, the release of ethyl sinapate into oils was also observed, which increased the ABTS, DPPH, and FRAP values of the analyzed samples by 36–62%, 10–44% and 218–259% after the first week and by 44–115%, 24–78% and 200–300% after the second week of accelerated storage compared to fresh oil. However, the absence of GPE film strips resulted in a decrease in the ABTS, DPPH and FRAP of oils by 1–15%, 3–21% and 11–31% and by 10–17%, 6–24% and 6–14% after the first and the second week of storage, respectively. This proves that the proposed packaging not only inhibits the oxidative process but also enhances the antioxidant potential of the analyzed oils. The highest increase in antioxidant potential was observed in terms of total polyphenol content, reaching approximately a 6-fold increase for oils stored with the active films after the first week, and between a 5–8-fold increase after the second week, compared to fresh oil. These results confirm the hypothesis that the addition of GPE film strips effectively increases the antioxidant properties of cold-pressed rapeseed oil. The observed increase after the first week also indicates the release of ethyl sinapate from the film strips into the oil, providing long-term protection. In contrast, oils without GPE film strips showed a decrease in antioxidant activity. Moreover, the use of GPE film strips inhibited oxidative processes compared to oils stored without this active film, demonstrating the dual role of the proposed packaging of cold-pressed rapeseed oil in enhancing its antioxidant potential and preventing oxidation. The substantial improvement in antioxidant features of the investigated oils highlights the need for further studies to identify which native antioxidants interact synergistically with ethyl sinapate and to determine which compounds undergo degradation during storage.

In conclusion, the developed gelatin/polyvinyl alcohol film strips fortified with ethyl sinapate demonstrate strong potential as an innovative packaging material for extending the shelf life and preserving the quality of cold-pressed rapeseed oil and other oxidation-sensitive edible oils.

## Data Availability

The original contributions presented in this study are included in the article. Further inquiries can be directed to the corresponding author.
